# Supplementary material for: Loss of a Functional Mitochondrial Pyruvate Carrier in Komagataella phaffii Does Not Improve Lactic Acid Production from Glycerol in Aerobic Cultivation
Source: Microorganisms. 2023 Feb 15;11(2):483. doi: 10.3390/microorganisms11020483 (PMC9967928; doi:10.3390/microorganisms11020483)
Supplement: Supplementary file 1 [file microorganisms-11-00483-s001.zip › microorganisms-2225995-supplementary.pdf]

# Supplementary Material

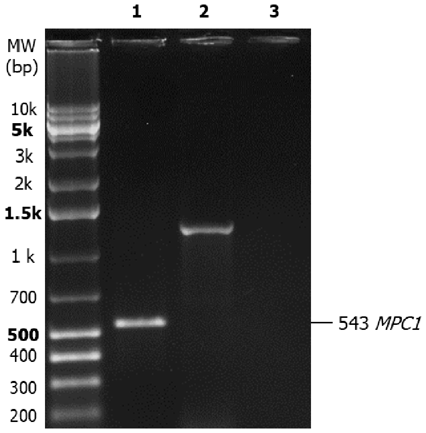


**Figure S1**. 1% agarose gel that confirmed the deletion of *Mpc1* in *K. phaffii* (GLp strain). 1 kb Plus DNA Ladder (Cellco, Brazil) is shown in the first column. **1**: Native *Mpc1* (543 bp) in non-transformed *K. phaffii* strain amplified with MPC1F and MPC1R. **2.** Cassette integration in *MPC1* locus amplified with RAD9F and pADH2R. **3.** Absence of *MPC1* gene in the transformed strain (GLpm).

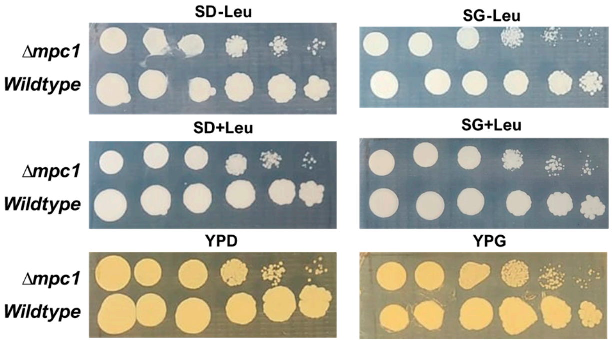


**Figure S2.** Spotting assay of *K. phaffii* strains in synthetic medium (S) with leucine (+Leu) or without leucine (−Leu) and in rich complex medium (YP). Plates were supplemented with 2% dextrose (D) or glycerol (G).

For the spotting assay, one colony of wildtype X-33 (Invitrogen) or Mpc1-deficient (X-33 Δmpc1; This work) was cultured overnight in 50 mL YPG. Cells were harvested by centrifugation, and cell pellet was washed with sterilized distilled water. Then, the culture was serially diluted to adjust optical densities to 0.5, 0.1, 0.02, 0.004, 0.0008, 0.00016. Ten microliters of each dilution were plated. Cells were cultured for 2 days.
